# Screening of Keratoconus Using Autokeratometer and Keratometer Keratoconus Index

**DOI:** 10.3390/diagnostics11112120

**Published:** 2021-11-15

**Authors:** Takashi Kojima, Naoki Isogai, Tomoya Nishida, Tomoaki Nakamura, Kazuo Ichikawa

**Affiliations:** 1 Department of Ophthalmology, Keio University School of Medicine, Shinanomachi 35, Shinjuku-ku, Tokyo 160-8582, Japan; 2 Nagoya Eye Clinic, Nagoya 456-0003, Japan; isogai@lasik.jp (N.I.); nishida@lasik.jp (T.N.); nic@bc5.so-net.ne.jp (T.N.); 3 Chukyo Eye Clinic, Nagoya 456-0032, Japan; ichikawa@chukyogroup.jp

**Keywords:** keratoconus, diagnosis, autokeratometer, astigmatism, keratometer keratoconus index (KKI)

## Abstract

The keratometer keratoconus index (KKI) is a diagnostic index for the risk of keratoconus calculated from autokeratometer test values. We partially modified the KKI equation and assessed it without limiting the target age and severity of keratoconus. This retrospective study included 179 eyes of 99 patients with keratoconus and 468 eyes from 235 normal controls. In the modified KKI, oblique astigmatism or against-the-rule astigmatism was defined as ≥1D astigmatism. KKI diagnostic power was analyzed in subgroups of <50 and ≥50-year-old patients, and at different keratoconus stages. Although the sensitivity of modified KKI was comparable with that of original KKI (92.7% vs. 95.5%), modified KKI specificity was significantly higher (79.7% vs. 68.6%) (*p* = 0.0001). Using the modified KKI, sensitivity reached 100% (4/4) and specificity, 63.5% (33/52), in ≥50-year-old patients, while overall sensitivity in keratoconus ≥stage 2 was 100% (30/30). In conclusion, the modified KKI proved to be effective in keratoconus screening at all stages. However, it should be noted that false-positive frequency is higher in ≥50-year-old patients.

## 1. Introduction

Keratoconus is a corneal ectatic disease that often occurs between 10–20 years of age. Although etiology of keratoconus is unknown, eye rubbing has been reported to be a risk factor for keratoconus development [1,2,3]. As the disease progresses, it causes a decrease in spectacle-corrected visual acuity due to an increase in irregular corneal astigmatism. It often requires rigid gas permeable (RGP) contact lenses for refractive correction, and in more severe cases, corneal transplantation.

In 2003, Wollensak et al. first reported that corneal crosslinking was effective in preventing the progression of keratoconus [4]. Since then, many studies have reported the efficacy and safety of corneal crosslinking [5,6,7,8,9,10,11,12,13,14,15,16,17,18,19,20,21,22]. Corneal crosslinking has been reported to be less effective in advanced keratoconus [23]. For this reason, early diagnosis of keratoconus is essential to maintain the visual function in keratoconus patients.

Keratoconus is generally diagnosed using slit lamp microscopy and a corneal shape analyzer. Localized corneal thinning, Fleisher’s ring, Vogt’s striae, and Munson’s sign are characteristic of keratoconus, but it is difficult to detect any of these findings using slit lamp microscopy in early keratoconus. On the other hand, corneal shape analyzers are effective in detecting early and suspected keratoconus. Evaluation of the corneal topography can allow the detection of subtle changes in the anterior surface of the cornea. In keratoconus eyes, the most prominent changes occur on the posterior surface of the cornea, while the anterior surface is smoothed by the corneal epithelium [24,25,26]. Thus, as corneal tomography can detect abnormalities on the posterior surface of the cornea and in corneal thickness distribution, it has been adopted for use in clinical practice in recent years.

Although corneal shape analyzers are installed in core regional eye hospitals and cornea specialists clinics, they are not available everywhere. Therefore, we reported a method to assess the risk of keratoconus with an autokeratometer, which is available in most institutions [27]. In this report, we created a keratometer keratoconus index (KKI) by combining three parameters: steep K, flat K, and whether or not the eye had with-the-rule astigmatism. We were able to detect keratoconus with 85% sensitivity and 86.7% specificity. Our previous study was limited to <50-year-old patients, and only early stage 1 keratoconus (Amsler–Krumeich classification) was considered.

In the KKI parameters, a dummy variable of 1 for with-the-rule astigmatism and 0 for non-with-the-rule astigmatism was used in the regression equation. However, in our preliminary study, when against-the-rule astigmatism and oblique astigmatism magnitude was small, some false positive results were observed. For this reason, we partially modified the KKI equation. Moreover, in actual clinical practice, KKI is likely to be used regardless of the age or severity of keratoconus. 

The purpose of this study was to apply the modified KKI to diagnose keratoconus regardless of age or keratoconus severity, and to evaluate KKI diagnostic power.

## 2. Materials and Methods

### 2.1. Patients and Study Design

One hundred seventy-nine eyes of 99 consecutive keratoconus and suspected keratoconus patients (68 males and 31 females, mean age 33.48 ± 15.41 years), who visited the Nagoya Eye Clinic from January 2019 to December 2020 and were tested with an autokeratometer (ARK-1s, Gamagori, Japan, NIDEK), were included in the study. We did not consider the time since the patient was diagnosed with keratoconus, but included consecutive cases that visited the clinic. During the same period, 468 eyes of 235 consecutive subjects (125 men and 110 women; mean age 37.55 ± 22.70 years) examined for refractive correction were included as normal controls. The control subjects included those who had no abnormalities on slit lamp biomicroscopy examination and corneal topography.

### 2.2. Diagnosis and Severity Classification of Keratoconus

Two cornea specialists diagnosed keratoconus through slit lamp microscopy and corneal topography (TMS-4, TOMEY, Nagoya, Japan). Keratoconus signs found in both slit lamp microscopy and corneal topography were classified as keratoconus, while signs found only in corneal topography were classified as suspected keratoconus. Forme fruste keratoconus was defined as an eye with normal corneal topography in the contralateral eye of the keratoconus. In this study, only keratoconus and suspected keratoconus were included in the keratoconus group. The severity of keratoconus was based on the Amsler–Krumeich classification. This study was approved by the Ethics Committee of Nagoya Eye Clinic (UMIN ID: 000036372). The study was conducted in accordance with the tenets of the Declaration of Helsinki. Because this was a retrospective study, an opt-out method for obtaining consent was approved by the ethics committee.

### 2.3. Modification of the Keratoconus Keratometer Index (KKI)

In this study, the same autokeratometer (ARK-1s) was used for all keratoconus patients and control subjects. The autokeratometer was calibrated according to international standards prior to measurement at the manufacturer. The KKI was derived using the parameters measured by the autokeratometer and the regression equation shown in our previous studies.

logit = 1.284 × steep K (diopter) − 0.618 × flat K (diopter) − 3.163 × (0: non-WTR astigmatism; 1: WTR astigmatism) − 28.662, KKI = exp(logit)/(1 + exp[logit])

WTR, with-the-rule.

In the previous KKI, the dummy variable was set to 0 for non-with-the-rule astigmatism and 1 for with-the-rule astigmatism. To reduce the number of false positives, the dummy variable was changed to 0:≥1 dpt of oblique or against-the-rule astigmatism, and 1: other; the KKI obtained by this change was defined as modified KKI.

### 2.4. Classification of Astigmatism Axis

Against-the-rule astigmatism was defined as 0° ≤ steep K axis < 30° or 150° ≤ steep K axis < 180°. Oblique astigmatism was defined as a 30° ≤ steep K axis < 60° or 120° ≤ steep K axis < 150°. With-the-rule astigmatism was defined as 60° ≤ steep K axis < 120°.

### 2.5. KKI vs. Modified KKI Diagnostic Power Comparison

The diagnostic sensitivity and specificity power were compared between KKI and modified KKI, and the same cutoff value (0.461) as previously reported was used for both KKI and modified KKI.

### 2.6. Comparison of Diagnostic Performance between Mild and Moderate/More Severe Keratoconus

Amsler–Krumeich classification stage 1 keratoconus was defined as the mild group, stage 2 and above as the moderate-to-severe group, and modified KKI sensitivity was compared between the groups.

### 2.7. Comparison of Diagnostic Performance between <50 and ≥50-Year-Old Patients

Control subjects and keratoconus patients were divided into two groups: <50 and ≥50-year-old, and sensitivity and specificity were compared between the groups.

### 2.8. Statistical Analyses

The Mann–Whitney U test was used to compare parameters between the control and keratoconus groups. Fisher’s exact test was performed to compare the sex and astigmatism distributions in the control and keratoconus groups. Similarly, Fisher’s exact test was used to compare the modified KKI sensitivity between the mild and moderate-to-severe keratoconus groups. SPSS (ver. 19, IBM, Endicott, NY, USA) was used for all statistical analyses, including multivariate analysis. *p*-value < 5% was considered statistically significant.

## 3. Results

### 3.1. Comparison of Background between Keratoconus and Control Groups

There was no significant difference in age between the two groups (*p* = 0.38), but there was a significant difference in sex distribution (*p* = 0.0088). Steep K and flat K were significantly higher in the keratoconus group than in the control group (steep K, *p* < 0.0001; flat K, *p* < 0.0001). The astigmatism distribution showed significantly more oblique and against-the-rule astigmatism in keratoconus eyes than in control eyes (*p* < 0.0001) (Table 1). As for severity, 89 eyes (49.7%) were classified as stage 1, 68 eyes (38.0%) as stage 2, 5 eyes (2.8%) as stage 3, and 17 eyes (9.5%) as stage 4.

When divided in <50 and ≥50-year-old groups, keratoconus patients showed no significant difference in corneal astigmatism distribution (*p* = 0.807), while normal controls did. In particular, the ≥50-year-old control group showed higher against-the-rule and oblique astigmatism rates than the <50-year-old group (Table 2). 

### 3.2. Modified KKI and KKI Diagnostic Power Comparison

**Figure 1:**
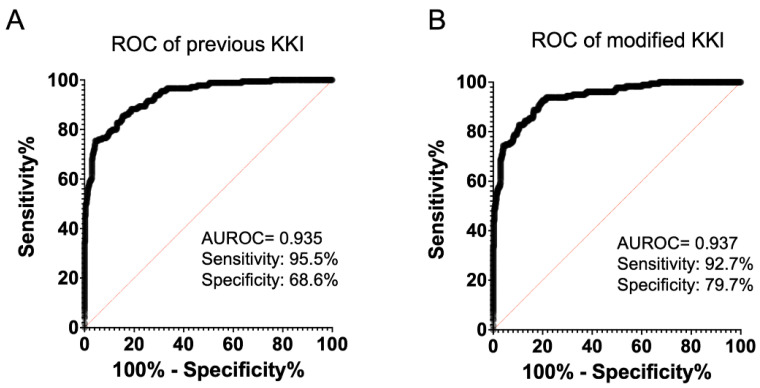
ROC curve analysis delineating the sensitivity and specificity of keratoconus screenings using an autokeratometer. ROC curves for each diagnostic parameter are shown for the KKI and modified KKI equation (**A**,**B**). AUROC, area under the ROC curve; KKI, keratometer keratoconus index; WTR, with-the-rule; CI, confidence interval.

KKI sensitivity and specificity were 95.5% (171/179) and 68.6% (321/468), respectively, while modified KKI sensitivity and specificity were 92.7% (166/179) and 79.7% (373/468), respectively (Figure 1). There was no significant difference in sensitivity between KKI and modified KKI (*p* = 0.37), but modified KKI had significantly higher specificity than KKI (*p* = 0.0001).

### 3.3. Evaluation of Modified KKI Diagnostic Performance in Mild and Moderate-to-Severe Keratoconus

In mild keratoconus eyes, sensitivity was 85.4% (76/89), while in moderate-to-severe keratoconus eyes, sensitivity reached 100% (90/90). The difference in diagnostic performance was statistically significant (*p* < 0.0001).

### 3.4. Comparison of Diagnostic Performance between <50 and ≥50 Years Old Groups

In the <50-year-old group, sensitivity was 92.9% (145/156), and specificity 83.3% (269/323). In the ≥50-year-old group, sensitivity was 100% (23/23) and specificity 68.9% (51/74). Sensitivity was significantly higher, and specificity significantly lower in the ≥50-year-old group (*p* < 0.0001; *p* = 0.0084, respectively).

## 4. Discussion

We have previously shown that KKI using autokeratometer parameters is useful in keratoconus screening [27]. In this report, the study was limited to early stage keratoconus and <50-year-old patients; however, in actual clinical situations, many cases exist outside this range. Therefore, we investigated the effects of age and severity of keratoconus on KKI diagnostic power in this study. In addition, we modified the astigmatism axis classification method used in the KKI because of false-positive results identified in the preliminary investigation. Diagnostic parameters of sensitivity and specificity were analyzed by comparing KKI and modified KKI results.

In the present study, sensitivity and specificity of the modified KKI were 92.7% and 79.7%, respectively. In our previous study, sensitivity and specificity were 85.0% and 86.7%, respectively. The higher sensitivity and lower specificity of the current study may be due to the age and severity restrictions of the previous sample.

In this study, we modified a parameter in the KKI calculated in our previous paper. In the autokeratometer, the astigmatic axis is calculated by elliptically approximating the corneal refractive power distribution. For this reason, the smaller the astigmatism, the greater the variation in the measured axis due to the influence of the tear film layer and other factors. Thus, in the modified KKI, the dummy variable for the astigmatic axis was defined as 0 for ≥1D of oblique or against-the-rule astigmatism, and 1 for other astigmatism. Consequently, sensitivity was similar, but specificity improved from 68.6 to 79.7%.

In our analysis, specificity was significantly lower in the ≥50-year-old group than in the <50-year-old group. When comparing astigmatism distribution between the <50 and ≥50-year-old groups, the percentage of with-the-rule astigmatism was found to be significantly smaller in the ≥50-year-old group, while the percentages of oblique and against-the-rule astigmatism were significantly larger. A previous study in Japan reported an increase in the rate of against-the-rule astigmatism ≥50-year-old people, [24] and our study seems to be consistent with these results. The decrease in specificity in the ≥50-year-old group may be due to a change in the astigmatic axis distribution [28].

Modified KKI sensitivity was 85.4% for stage 1 (mild keratoconus) vs. 100% for stage 2 and above (moderate keratoconus). The parameters used for KKI were steep K, flat K, and astigmatism axes. The more severe the keratoconus, the greater the difference in both steep K and flat K from normal eyes; therefore, the present results seem reasonable.

Sensitivity rates of 85.4% for stage 1 and 92.7% for all stages are acceptable for the clinical use of modified KKI. However, specificity was 83.3% even when limited to <50-year-old patients, which means that approximately 17% of normal cases are false positives. This may pose a problem in clinical practice. Keratoconus has been associated with atopic dermatitis, Down syndrome, sleep apnea syndrome, and eye rubbing [29,30,31,32,33]. Future research should aim to further increase KKI specificity by considering other parameters and contributing factors.

This study had several limitations. Firstly, this study was carried out retrospectively and included only patients who were diagnosed with keratoconus or suspected keratoconus at one time point. In order to confirm the utility of the modified KKI in the future, it is necessary to evaluate the KKI over time as a prospective study and to examine when keratoconus can be detected. Secondly, the sample size of the disease group was smaller than that of the control group. Since keratoconus has a large variability from case to case, it may be necessary to confirm this by examining a larger number of cases. Thirdly, corneal refractive power and corneal astigmatism have been reported to vary with ethnicity [34,35,36,37,38,39,40,41]. Since all of the participants in this study were Japanese, further studies including other ethnicities are needed. Fourthly, only one model of autokeratometer was used. Although autokeratometer was calibrated according to international standards in the current study, it may be necessary to verify whether modified KKI is effective for other models as well.

## 5. Conclusions

Modified KKI is effective in mild to severe keratoconus screening. It may be a useful tool in clinical practice, although caution is required when examining patients older than 50 years, as its diagnostic power decreases.

## Figures and Tables

**Table 1:** Control subjects and keratoconus patients demographic information.

	Keratoconus Group	Control Group	*p* Value
Number (eyes)	179	468	
Age (years old)	33.48 ± 15.41	37.55 ± 22.70	0.3794
	(10–79)	(7–86)	
Sex (male: female)	68:31	125:110	0.0088
Autokeratometer parameters			
Steep K (D)	48.51 ± 4.63	43.99 ± 3.6	<0.0001
	(42.03–72.42)	(40.04–51.29)	
Flat K (D)	46.56 ± 4.08	42.77 ± 1.50	<0.0001
	(38.09–61.93)	(38.70–48.84)	
Astigmatism (D)	3.92 ± 2.76	1.35 ± 1.05	<0.0001
	(0.12–12.36)	(0.06–7.2)	
Autokeratometer astigmatism axis			
WTR (eyes, %)	86	367	<0.0001
	(48.0%)	(78.4%)	
ATR (eyes, %)	41	58	
	(22.9%)	(12.4%)	
Oblique (eyes, %)	52	43	
	(29.1%)	(9.2%)	

K, keratometric power; D, diopter; WTR, with the rule; ATR, against the rule.

**Table 2:** Comparison of corneal astigmatism axis distribution.

	Keratoconus Group		Control Group	
	<50 y.o.	≥50 y.o.	<50 y.o.	≥50 y.o.
WTR (eyes, %)	76	10	293	74
	(48.7%)	(43.5%)	(90.7%)	(51.0%)
ATR (eyes, %)	36	5	12	46
	(23.1%)	(21.7%)	(3.7%)	(31.7%)
Oblique (eyes, %)	44	8	18	25
	(28.2%)	(34.8%)	(5.6%)	(17.2%)
**Difference of Distribution**			***p* Value**	
Keratoconus group (<50) vs. Keratoconus group (≥50)			0.807	
Control group (<50) vs. Control group (≥50)			<0.0001	
Keratoconus group (<50) vs. Control group (<50)			<0.0001	
Keratoconus group (<50) vs. Control group (≥50)			0.0477	
Keratoconus group (≥50) vs. Control group (<50)			<0.0001	
Keratoconus group (≥50) vs. Control group (≥50)			0.136	

y.o., years old; WTR, with the rule; ATR, against the rule.

## Data Availability

The data presented in this study are available upon request from the corresponding author. The data are not publicly available according to the ethics committee indications.
